# *Staphylococcus aureus* Sb‐1 Bacteriophage Alters Frequency and Subset Distribution of Human Innate Lymphoid Cells

**DOI:** 10.1111/all.70212

**Published:** 2026-02-03

**Authors:** Ramazan Rozumbetov, Ismail Ogulur, Anja Heider, Anna Globinska, Nina Chanishvili, Elene Kakabadze, Marina Goderdzishvili, Nino Grdzelishvili, Nata Bakuradze, Moelong Yu, Tamara Aripova, Adolat Ismailova, Nikolaos Papadopoulos, Mubeccel Akdis, Cezmi A. Akdis

**Affiliations:** ^1^ Swiss Institute of Allergy and Asthma Research (SIAF), Davos University of Zurich Zurich Switzerland; ^2^ Institute of Immunology and Human Genomics Tashkent Uzbekistan; ^3^ The Eliava Institute of Bacteriophage, Microbiology & Virology Tbilisi Georgia; ^4^ Eliava BioPreparations Ltd Tbilisi Georgia; ^5^ Allergy Department, National Kapodistrian University of Athens, Athens, Greece & Division of Infection, Immunity & Respiratory Medicine University of Manchester Manchester UK

**Keywords:** bacteriophage, immune system, innate lymphoid cells

To the Editor,

Bacteriophages (phages), viruses that infect bacteria, are the most abundant biological entity on Earth [1]. With the rise of multidrug‐resistant bacteria, interest in phage research has been rekindled over the last decades. Colonizing our body along with bacteria, bacteriophages modulate the microbiota in all our mucosal surfaces, such as the lung, vagina, skin, oral, and intestinal tissues [2]. Various phages induce IL‐10, IL‐1β, IL‐6, TNFα, CXCL1, CXCL5, and expression of immune‐related genes [3]. Innate lymphoid cells (ILCs) populate the submucosal area in the barrier organs and play a role in various pathologies, and the interaction between ILCs and bacteriophages may play a role in modifying the immunologic profile of ILCs [4]. While the phage–bacteria and bacteria–immune system interactions are well studied, the crosstalk of phage and immune system is yet to be revealed, and understanding of which may pave the way for new therapeutics in the future.

We characterized ILC responses to *S. aureus* Sb‐1 phage in cultures of peripheral blood mononuclear cells (PBMCs) of healthy individuals. For control, bacterial lysate was purified with the same method as the phage, and used to evaluate the effect of bacterial residues (Figure S1). Flow cytometry results revealed that stimulation of PBMC with the Sb‐1 phage at a concentration of 10^9^ PFU/mL decreases the number of all ILC subsets, as well as purified *S. aureus* lysate; however, after phage stimulation, the total ILC number dropped to significantly lower levels (Figure 1 and Figure S1). The anti‐inflammation and immunomodulatory effects of phages [3] could potentially affect ILC survival indirectly through changes in cytokine milieu or immune signaling pathways. Regarding their percentage in total ILCs, there was a dose‐dependent increase in ILC1 and a decrease in ILC3 (Figure 1 and Figure S2). After stimulation with purified *S. aureus* lysate, the tendency of the findings was the same, but the extent of the phage effect was more pronounced (Figure S1). This finding could be attributed to the functional plasticity of ILCs, because of the previous demonstration of a switch from ILC3 to ILC1 [5]. Both NKp44‐ and NKp44+ ILC3 numbers decreased in cultures from 24 h to 72 h. These results suggest that Sb‐1 promotes an increase in the frequency of ILC1 in a dose‐dependent manner; however, confirmation of whether the phage effect is direct or indirect on ILC would require further experiments.

**FIGURE 1:**
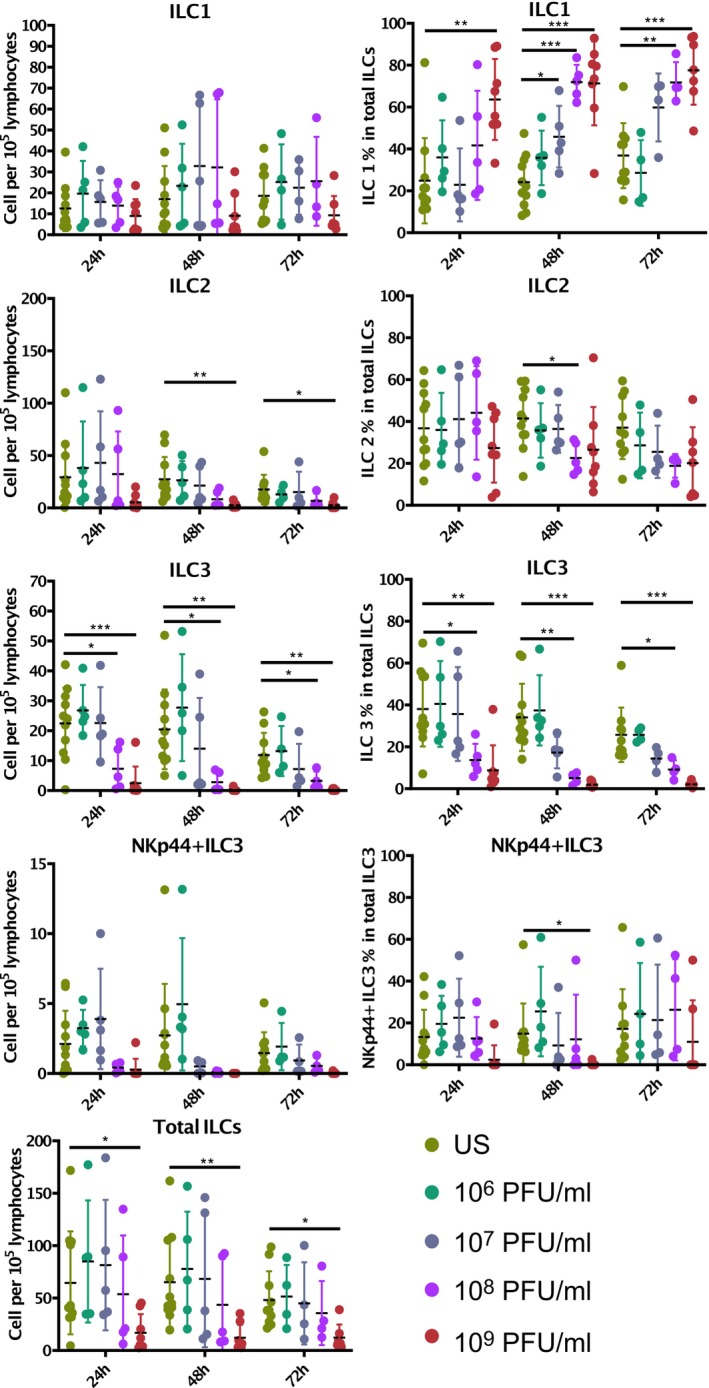
Number and frequency of ILC subsets change in response to phage stimulation. ILC1 was identified as CRTH2‐ CD117‐, ILC2 as CRTH2+ CD117‐, and ILC3 as CRTH2‐ CD117+. NKp44‐positive ILC3 and CD69‐positive ILCs were determined. ILC1, ILC2, ILC3, and NKp44+ ILC3 within total ILCs (Lin‐ CD127+ CD161+) after stimulation of freshly isolated PBMC with 10^6^, 10^7^, 10^8^, and 10^9^ PFU/mL *S. aureus* Sb‐1 phage for 24 h, 48 h, and 72 h (*N* > 4). US, unstimulated.

CD69, an early activation marker of ILCs, was used to study the immunomodulating effect of the Sb‐1 phage on ILC subsets (Figure 2 and Figure S3). Progression of CD69+ subsets of ILCs is clearly seen in Figure S3. In response to stimulation with the phage, the number and percentage of activated ILC1 and ILC2 increased, with a concomitant decrease of absolute numbers of activated ILC3 per total lymphocytes in bacteriophage doses of 10^8^ and 10^9^ phage‐forming units (PFU)/mL (Figure 2). Activated ILC populations were visualized using a t‐distributed stochastic neighbor embedding (t‐SNE) algorithm (Figure S4). In consistency with the numbers in Figure 2, the t‐SNE results revealed a downregulation in CD69+ ILC3 population and upregulation of CD69+ ILC1 and CD69+ ILC2 populations after stimulation with phage (Figure S4).

**FIGURE 2:**
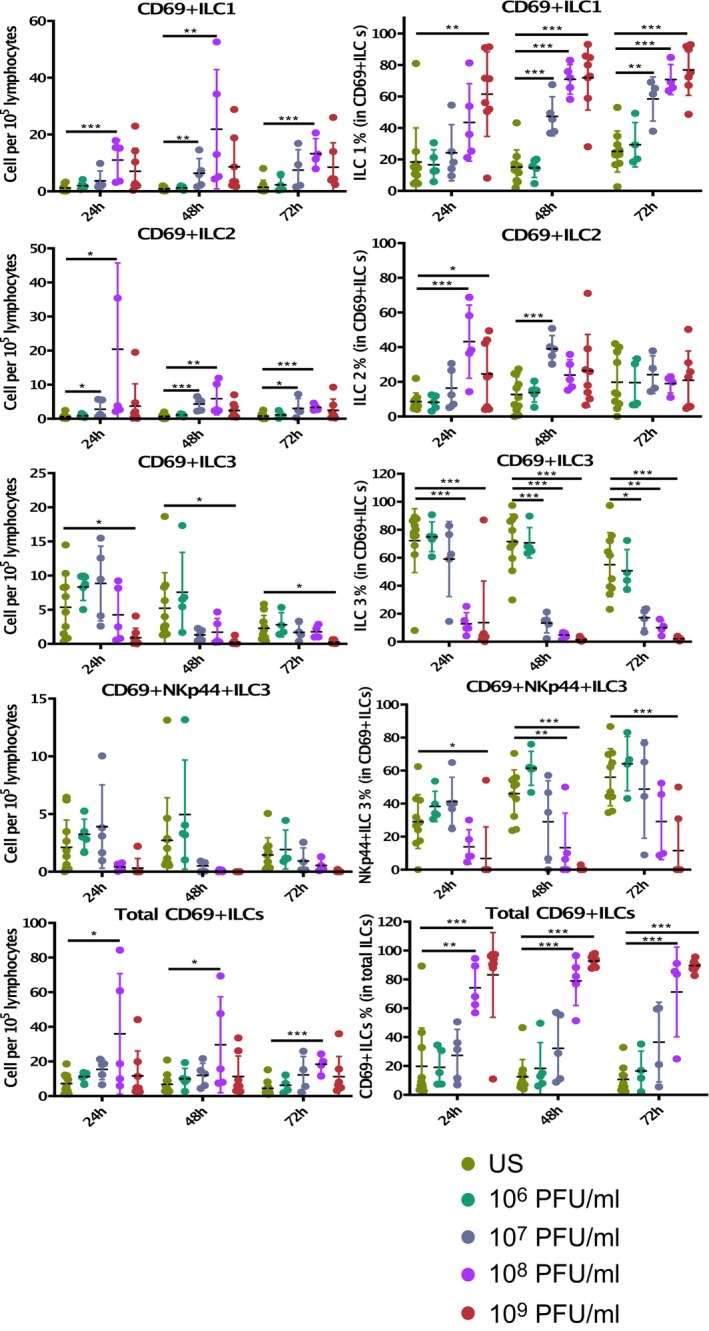
Phages activate ILC1 and ILC2. Number and frequency of activated ILC1, ILC2, ILC3, and NKp44+ ILC3 within total ILCs (Lin‐ CD127+ CD161+) after stimulation of freshly isolated PBMC with 10^6^, 10^7^, 10^8^, and 10^9^ PFU/mL *S . aureus* Sb‐1 phage for 24 h, 48 h, and 72 h (*N* > 4). US, unstimulated.

In addition, we investigated the role of *S. aureus* Sb‐1 phage on the expression of specific proinflammatory and immunoregulatory proteins in PBMC. Accordingly, the expression of 29 immune‐related proteins was evaluated in the supernatants, namely HNMT, IL10, PRDX5, KLRD1, IRAK4, MILR1, IL12RB1, CD83, AREG, CCL11, TRIM21, CLEC4C, CLEC7A, LILRB4, SPRY2, CLEC6A, PRDX1, IL5, STC1, PPP1R9B, DCTN1, ZBTB16, SH2B3, MGMT, TRAF2, DAPP1, SPRK2, IRAK1, and SPRY2. The immunomodulatory proteins IL‐10, IL‐6, CCL‐11, AREG and LILRB4 were markedly elevated at all three timepoints (Figure S5). Induction of IL‐10, a prominent immunosuppressive cytokine, and pleotropic cytokine IL‐6, which has both pro‐ and anti‐inflammatory activities, has been previously observed in response to phage stimulation [6]. These cytokines regulated by phages may play substantial roles in inflammation and immune tolerance [7]. Also, Allergin‐1, an inhibitory receptor that suppresses anaphylaxis in mice, was downregulated (Figure S5), and it is worth noting that a similar result was previously observed [8]. A maturation marker of dendritic cells, CD83, was upregulated in response to phage (Figure S5), as previously reported [3].

In conclusion, we demonstrate for the first time that *S. aureus* Sb‐1 phage can regulate human ILCs. Our data reported here open a new window for phage research and interaction between the immune system and microbiota. We show that *S. aureus* Sb‐1 phage activates and stimulates the proliferation of particularly ILC1 but also ILC2. A plethora of cytokines and immune‐response or inflammation‐related proteins are regulated, suggesting the contribution of phages to immune tolerance and inflammation. Moreover, phage exposure may alter cytokine networks that govern ILC plasticity, potentially shifting inflammatory versus regulatory ILC states and thereby influencing disease severity or modifying the immunological outcomes of phage therapy, highlighting the importance of accounting for host immune modulation in clinical applications.

## Author Contributions

R.R., N.C., and C.A.A. conceived and designed the study, and planned the experiments. I.O., A.H., A.G., E.K., M.G., N.G., N.B., M.Y., T.A., and A.I. helped with the experiments. R.R. and C.A.A. wrote the manuscript. N.C., N.P., M.A., and C.A.A. reviewed the manuscript.

## Funding

This work was supported by European Union Horizon.

## Conflicts of Interest

I.O. is chair of the EAACI Epithelial Cell Biology Working Group. M.A. has received research grants from the Swiss National Science Foundation, Bern; research grant from Stanford University; Leading House for the Latin American Region, Seed Money Grant. She is the Scientific Advisory Board member of Stanford University Sean Parker Asthma Allergy Center, CA; Advisory Board member of LEO Foundation Skin Immunology Research Center, Copenhagen; and Scientific Co‐Chair of World Allergy Congress (WAC) Istanbul, 2022, Scientific Programme Committee Chair, EAACI. C.A.A. has received research grants from the Swiss National Science Foundation, European Union (EU CURE, EU Syn‐Air‐G), Novartis Research Institutes (Basel, Switzerland), Stanford University (Redwood City, Calif), Seed Health (Boston, USA), and SciBase (Stockholm, Sweden); is the Co‐Chair for EAACI Guidelines on Environmental Science in Allergic diseases and Asthma; is on the Advisory Boards of Sanofi/Regeneron (Bern, Switzerland, New York, USA), Stanford University Sean Parker Asthma Allergy Center (CA, USA), Novartis (Basel, Switzerland), Glaxo Smith Kline (Zurich, Switzerland), Bristol‐Myers Squibb (New York, USA), Seed Health (Boston, USA), and SciBase (Stockholm, Sweden); and is the Editor‐in‐Chief of Allergy. R.R., A.H., A.G., N.C., E.K., M.G., N.G., N.B., M.Y., T.A., A.I., and N.P. declare no relevant conflicts of interest.

## Supporting information


**Table S1:** List of antibodies used in flow cytometry.
**Figure S1:** PBMCs stimulation with Sb‐1 phage and purified *S. aureus* lysate.
**Figure S2:** Gating strategy for ILC subsets.
**Figure S3:** Activation of ILC subsets in response to phage stimulation.
**Figure S4:** all70212‐sup‐0001‐AppendixS1.pdf. *t*‐Distributed Stochastic Neighbor Embedding (tSNE) technique applied on ILCs.
**Figure S5:** Volcano plots of cytokine and pro‐ and anti‐inflammatory protein levels.

## Data Availability

The data that support the findings of this study are available from the corresponding author upon reasonable request.
