# Supplementary material for: Staphylococcus aureus Sb‐1 Bacteriophage Alters Frequency and Subset Distribution of Human Innate Lymphoid Cells
Source: Allergy. 2026 Feb 3;81(8):2942–6. doi: 10.1111/all.70212 (PMC13466002; doi:10.1111/all.70212)
Supplement: Supplementary file 1 [file ALL-81-2942-s001.pdf]

Staphylococcus aureus Sb-1 bacteriophage alters frequency and subset distribution of human innate lymphoid cells

Ramazan Rozumbetov[a,b], Ismail Ogulur[a], Anja Heider[a], Anna Globinska[a], Nina Chanishvili[c], Elene Kakabadze[c], Marina Goderdzishvili[c,d], Nino Grdzelishvili[c], Nata Bakuradze[c], Moelong Yu[a], Tamara Aripova[b], Adolat Ismailova[b], Nikolaos Papadopoulos[e], Mubeccel Akdis[a], Cezmi A. Akdis[a*]

[a] Swiss Institute of Allergy and Asthma Research (SIAF), Davos, University of Zurich, Switzerland

[b] Institute of Immunology and human genomics, Tashkent, Uzbekistan

[c] The Eliava Institute of Bacteriophage, Microbiology & Virology, Tbilisi, Georgia

[d] Eliava BioPreparations Ltd, Tbilisi, Georgia

[e] Allergy Dpt, 2nd Pediatric Clinic, National Kapodistrian University of Athens, Athens, Greece & Division of Infection, Immunity & Respiratory Medicine, University of Manchester, Manchester, UK

* Cezmi A. Akdis, Swiss Institute of Allergy and Asthma Research (SIAF), University of Zurich, Herman-Burchard-Strasse 9, CH-7265 Davos Wolfgang, Switzerland, e-mail: akdisac@siaf.uzh.ch

Online Supporting Information

Methods

Freshly isolated PBMC from healthy donors were incubated with 10^6, 10^7, 10^8 and 10^9 PFU/mL Staphylococcus aureus (*S. aureus*) Sb-1 bacteriophage for 24, 48 and 72 hours in cRPMI medium at 37 °C and 5% C02. PBMC were then collected and stained with fluorescent-conjugated antibodies. Classical immune cell lineage markers were stained with FITC and excluded from analysis (Figure S1: gating strategy). Total ILCs were determined as CD161 and CD127 positive cells. In order to discriminate between ILC subsets, CD117 and CRTH2 markers were used.

Flow cytometric analysis

The following antibodies against human proteins were used to determine the expression of surface markers. Fluorescein isothiocyanate-conjugated anti-CD1a (HL149), anti-CD11c (3.9), anti-CD34 (581), anti-CD94 (DX22), anti-CD123 (6H6), anti-CD303 (201A), anti-FcεRIa (AER-37), anti-CD3 (UCHT1), anti-CD4 (RPA-T4), anti-CD8a (RPA-T8), anti-CD14 (HCD14), anti-CD16 (B73.1) and anti-CD19 (HIB19) antibodies were used for detecting lineage markers. Phycoerythrin-conjugated (PE) anti-NKp44 (P44-8), PE-CF594-conjugated anti-CD117, peridinin-chlorophyll proteins (PerCP) Cy5.5-conjugated anti-CD161 (HP3G10), PE/Cy7-conjugated anti-CD127 (HIL-7RM21), Alexa Fluor (AF) 647-conjugated anti-CRTH2 (BM16), AF700-conjugated anti-CD25 (M-A251), Brilliant Violet (BV) 421-conjugated anti CD69 (FN50) and BV510-conjugated anti-CD45 (HI30) were used to discriminate ILC subsets (Table S1). Flow cytometry was performed on FACSAria II (BD Biosciences, Franklin Lakes, NJ).

Phage propagation and purification steps

Staphylococcus aureus Sb-1 phage was incubated in 500 mL flask with $1*10^7$ *Staphylococcus aureus* subsp. *aureus* Rosenbach (ATCC 6538) until the culture turned clear. After propagation of phages in the bacterial culture, the suspension was centrifuged for 30 min, 4 °C, at 4122 xg. The supernatant was then filtered through a 0.22 μm membrane and centrifuged for 1 h, 8 °C, at 25000 x g. Afterwards, the supernatant was discarded and the pellet resuspended in 500 μL of PBS. After ultracentrifugation the LPS concentration was 0.06 EU/mL as measured using a PierceTM Chromogenic Endotoxin Quant Kit (Thermo Fisher Scientific).

Human PBMC stimulation.

Human PBMC from healthy donors were isolated by gradient centrifugation and stimulated with 10^6, 10^7, 10^8 and 10^9 PFU/mL *Staphylococcus aureus* subsp. *aureus* Rosenbach (ATCC 6538) Sb-1 bacteriophage for 24 h, 48 h and 72 h in cRPMI medium at 37 °C, 5% CO_2. As a control, purified bacteria lysate was used. $1*10^7$ *S.aureus* was sonicated for 30 min and filtered through a 0.22 µm membrane and centrifuged for 1 h, 8 °C, at 25000 x g. The supernatant was then discarded and the pellet resuspended in 500 µl of PBS.

t-Distributed Stochastic Neighbor Embedding

A t-Distributed Stochastic Neighbor Embedding (tSNE) algorithm was applied to visualize ILC subsets using a tSNE plugin in Flowjo 10.6.1. Iterations: 1000. Perplexity: 20. Eta: 200. Iterations per frame: 5 (n>4). US, unstimulated. Iterations: 1000. Perplexity: 20. Eta: 200. Iterations per frame: 5.

Statistical analysis

Data analysis was performed with Prism Version 7.0e software (GraphPad). Unpaired T test. Statistical significance determined using the Holm-Sidak method, setting alpha as 0.05.

Table S1. List of antibodies used in flow cytometry

Marker	Dye	Company	Isotype	Clone	Cat.#	Volume per 10 mln PBMCs
CD1a	FITC	Biolegend (Lucerna-Chem AG)	M IgG1k	HI149	300104	2.5 ul
CD11c	FITC	Biolegend (Lucerna-Chem AG)	M IgG1k	3,9	301604	
CD34	FITC	Biolegend (Lucerna-Chem AG)	M IgG1k	581	343504	
CD94	FITC	Biolegend (Lucerna-Chem AG)	M IgG1k	DX22	305504	
CD123	FITC	Biolegend (Lucerna-Chem AG)	M IgG1k	6H6	306014	
CD303	FITC	Biolegend (Lucerna-Chem AG)	M IgG2ak	201A	354208	
FceR1a	FITC	Biolegend (Lucerna-Chem AG)	M IgG2bk	AER-37	334608	
CD3	FITC	Biolegend (Lucerna-Chem AG)	M IgG1k	UCHT1	300406	
CD4	FITC	Biolegend (Lucerna-Chem AG)	M IgG1k	RPA-T4	300506	
CD8a	FITC	Biolegend (Lucerna-Chem AG)	M IgG1k	RPA-T8	301006	
CD14	FITC	Biolegend (Lucerna-Chem AG)	M IgG1k	HCD14	325604	
CD16	FITC	Biolegend (Lucerna-Chem AG)	M IgG1k	B73.1	360716	
CD19	FITC	Biolegend (Lucerna-Chem AG)	M IgG1k	HIB19	302206	
NKp44 (CD 336)	PE	Biolegend (Lucerna-Chem AG)	M IgG1k	P44-8	325108	5
CD117 (c-Kit)	PE-CF594	BD Biosciences	M IgG1k	YB5.B8	562407	7,5
CD161	PerCPCy5.5	Biolegend (Lucerna-Chem AG)	M IgG1k	HP-3G10	339908	5
CD127	PE/Cy7	BD	M IgG1k	HIL-712-M12	560822	5
CRTH2	AF647	BD Biosciences	Rat, IgG2a,k	BM16	558042	5
CD25	AF700	BD	M IgG1k	M-A251	561398	2,5
CD69	BV421	Lucerna-Chem AG	M IgG1k	FN50	310930	5
CD45	V510	Biolegend (Lucerna-Chem AG)	M IgG1k	HI30	304036	2,5
Viability dye AF780	APC-Cy7	eBiosciences			65-0865	1 ul/mL

Figure S1

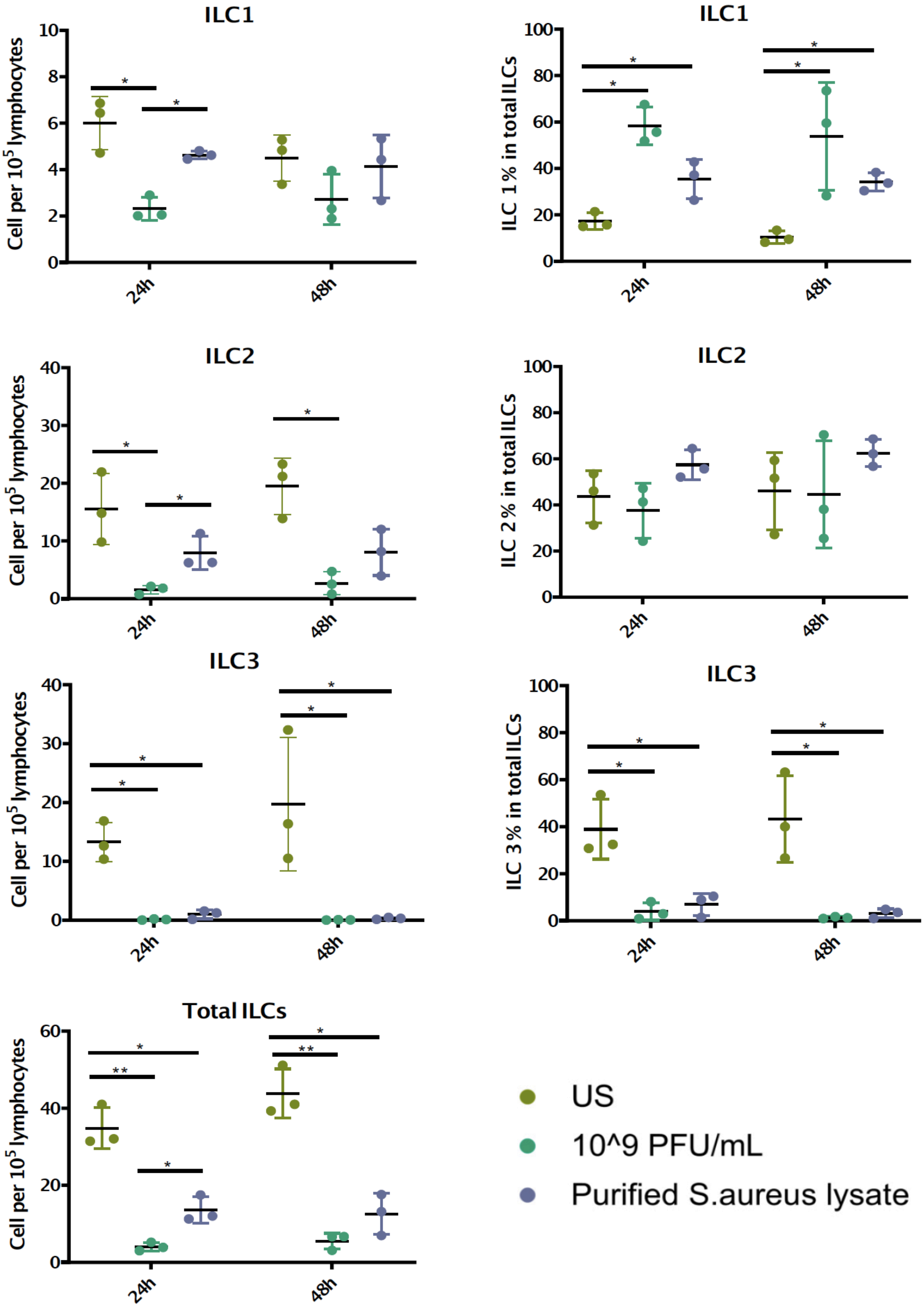

Figure S1. PBMCs stimulation with Sb-1 phage and purified S. aureus lysate. Number and frequency of ILC subsets change in response to phage and purified S. aureus lysate stimulation, which was used as a control to exclude the effect of bacterial residues. ILC1 was identified as CRTH2- CD117-, ILC2 as CRTH2+ CD117- and ILC3 as CRTH2- CD117+. ILC1, ILC2, ILC3

within total ILCs (Lin- CD127+ CD161+) after stimulation of freshly isolated PBMC with 10^9 PFU/mL S. Aureus Sb-1 phage for 24 h and 48 h (n=3). US, unstimulated.

Figure S2

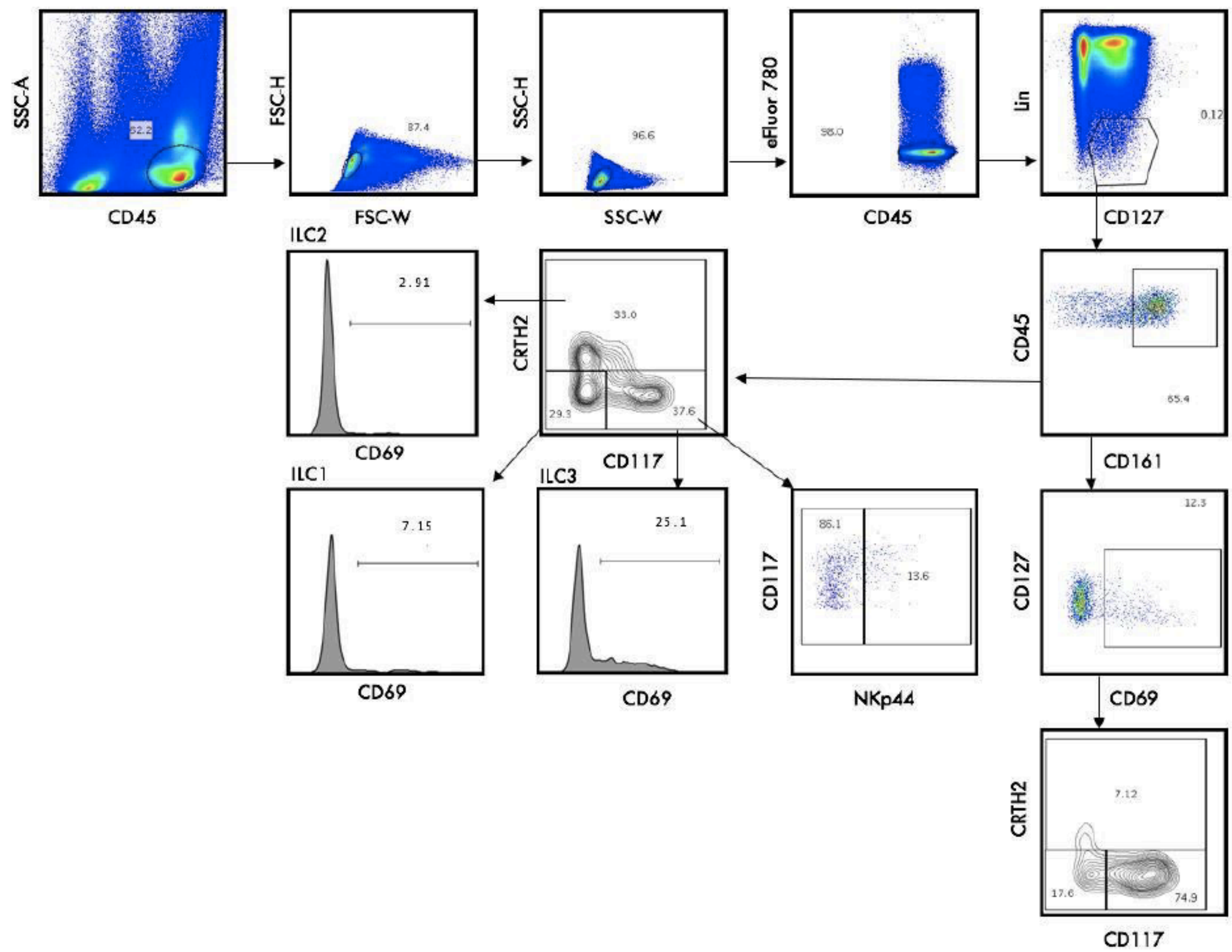

Figure S2. Gating strategy for ILC subsets. Lymphocytes were gated based on SSC-A and CD45+. Doublet and non-viable cells were gated out by further gates. Total ILCs were identified as Lin- CD127+ CD161+. ILC1, ILC2, ILC3 were determined on the basis of CRTH2 and CD117 expression within total ILCs. ILC3 subsets were identified by CD117 and NKp44+ expression. Activated ILCs were discriminated based on the expression of CD69+ (n>4). US, unstimulated.

Figure S3

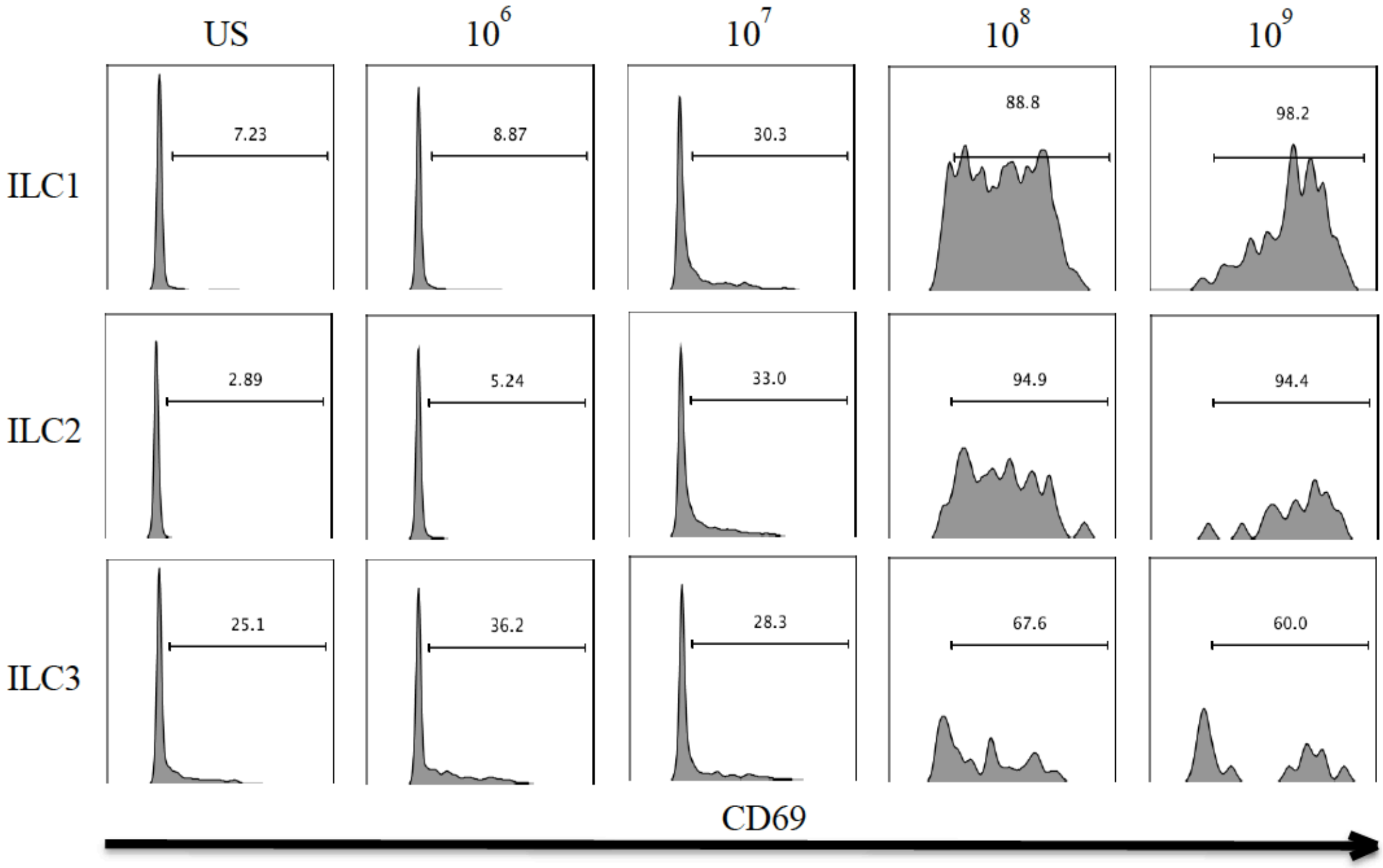

Figure S3. Activation of ILC subsets in response to phage stimulation. Histograms showing expression of activation marker, CD69, by ILC subsets after 24 hours of stimulation with different phage concentrations (10^6, 10^7, 10^8 and 10^9 PFU/mL). Activation of ILC1 increase in a dose-dependent manner (n>4). US, unstimulated.

Figure S4

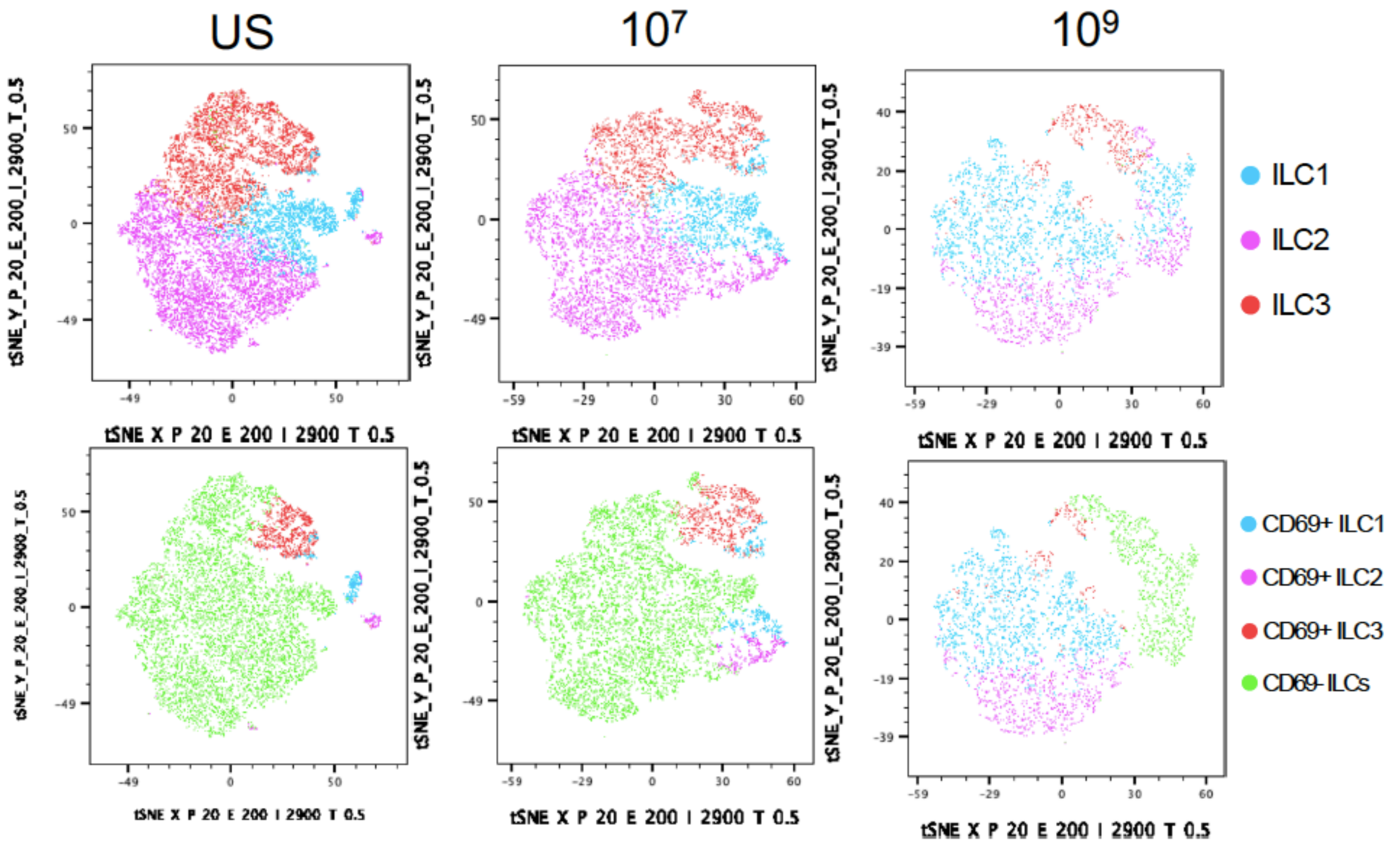

Figure S4. t-Distributed Stochastic Neighbor Embedding (tSNE) technique applied on ILCs. Freshly isolated PBMC of healthy donors were stimulated with 10^7 and 10^9 PFU/mL *S. aureus* Sb-1 phage for 24 hours. Activated ILC1 and ILC2 increase in response to 10^9 PFU/ml phage. Total ILC populations of unstimulated, 107 and 109 concentrations were connected into seperate groups and tSNE technique was applied. Iterations: 1000. Perplexity: 20. Eta: 200. Iterations per frame: 5 (n>4). US, unstimulated.

Figure S5

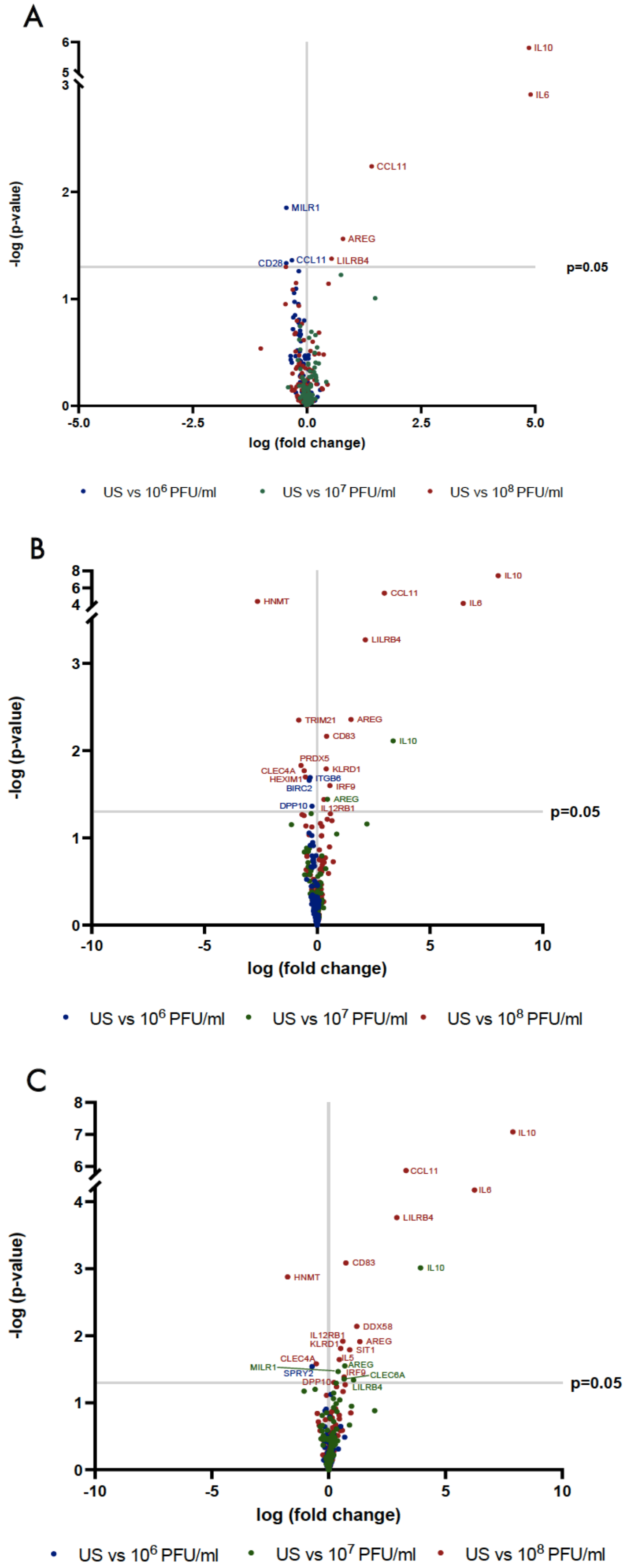
A
-log (p-value)
log (fold change)
p=0.05
IL10
IL6
CCL11
MILR1
AREG
CD28
CCL11
LILRB4
US vs 10^6 PFU/ml
US vs 10^7 PFU/ml
US vs 10^8 PFU/ml
B
-log (p-value)
log (fold change)
p=0.05
IL10
HNMT
CCL11
IL6
LILRB4
TRIM21
AREG
CD83
IL10
PRDX5
CLEC4A
KLRD1
HEXIM1
ITGB8
BIRC2
IRF9
AREG
DPP10
IL12RB1
US vs 10^6 PFU/ml
US vs 10^7 PFU/ml
US vs 10^8 PFU/ml
C
-log (p-value)
log (fold change)
p=0.05
IL10
CCL11
IL6
LILRB4
CD83
IL10
HNMT
DDX58
IL12RB1
KLRD1
AREG
SIT1
CLEC4A
IL5
MILR1
AREG
SPRY2
IRF9
CLEC6A
DPP10
LILRB4
US vs 10^6 PFU/ml
US vs 10^7 PFU/ml
US vs 10^8 PFU/ml

Figure S5. Volcano plots of cytokine and pro- and anti-inflammatory protein levels. Supernatants from incubation of PBMCs with and without phage were collected after 24 (A), 48 (B) and 72 (C) hours, and studied via Proximity Extension Assay (n>4). US, unstimulated.
